# Evaluation of the FitBark Activity Monitor for Measuring Physical Activity in Dogs

**DOI:** 10.3390/ani11030781

**Published:** 2021-03-11

**Authors:** Jessica Colpoys, Dean DeCock

**Affiliations:** 1 Department of Agricultural Science, Truman State University, Kirksville, MO 63501, USA; 2 Department of Statistics, Truman State University, Kirksville, MO 63501, USA; decock@truman.edu

**Keywords:** animal behavior, animal welfare, dog, canine, pet, accelerometer, activity monitor, FitBark, wearable technology, validation

## Abstract

**Simple Summary:**

Altered activity in a dog can be an early indicator of health and welfare concerns. Accelerometers, a type of activity monitor, are being used more frequently in dogs and may be a simple way for owners and veterinarians to monitor a dog’s changing health and welfare needs. However, there are few peer-reviewed studies evaluating the accuracy of these devices. Therefore, this study evaluated the accuracy of the FitBark 2 accelerometer (FitBark) by comparing activity data recorded using the FitBark to dog physical activity recorded using video analysis of dog step count. Dog step count and FitBark activity were highly correlated when the dogs were exploring a room off-leash and when they were interacting with their owner. However, when the dogs were being walked on a leash, low correlations between step count and FitBark activity were observed. In conclusion, the FitBark is a valid tool for tracking off-leash activity in dogs; however, more work should be done to identify the best method of tracking activity in on-leash situations.

**Abstract:**

Accelerometers track changes in physical activity which can indicate health and welfare concerns in dogs. The FitBark 2 (FitBark) is an accelerometer for use with dogs; however, no studies have externally validated this tool. The objective of this study was to evaluate FitBark criterion validity by correlating FitBark activity data to dog step count. Dogs (*n* = 26) were fitted with a collar-mounted FitBark and individually recorded for 30 min using a three-phase approach: (1) off-leash room explore; (2) human–dog interaction; and (3) on-leash walk. Video analysis was used to count the number of times the front right paw touched the ground (step count). Dog step count and FitBark activity were moderately correlated across all phases (r = 0.65, *p* < 0.001). High correlations between step count and FitBark activity were observed during phases 1 (r = 0.795, *p* < 0.001) and 2 (r = 0.758, *p* < 0.001), and a low correlation was observed during phase 3 (r = 0.498, *p* < 0.001). In conclusion, the FitBark is a valid tool for tracking physical activity in off-leash dogs; however, more work should be done to identify the best method of tracking on-leash activity.

## 1. Introduction

Changes in physical activity level can be indicative of health and welfare concerns in dogs. Below-normal activity levels can be an indicator of animal sickness [1,2], impaired dog mobility [3], and pain [4]. Above-normal activity levels can also signal health concerns, such as canine pruritus [5]. Additionally, physical activity levels can be an indicator of the amount of rest dogs are getting, as poor sleep quality can relate to welfare concerns in dogs [6]. In order to provide individualized animal treatment, it is important for dog owners, veterinarians, researchers, and other animal professionals to know the normal activity level for an individual dog, as deviations in either direction can serve as early indicators of health concerns. Thus, an accurate and accessible activity monitor for dogs can help differentiate an individual dog’s normal activity level and activity abnormalities.

Accelerometers, a type of activity monitor, are non-invasive tools used to track changes in acceleration [7,8]. As technology has advanced, activity monitors have become smaller and can provide automated feedback through integration with mobile devices [9]. Modern activity monitors serve as a more feasible option for research monitoring dog activity than live or video-recorded behavioral observations, which can be time-consuming and often require expensive software. While wearable technology is increasingly popular for humans [10], it is also becoming popular for pets, and multiple devices marketed toward pets are available [11]. A study evaluating an owner’s use of activity monitors for dogs determined that these devices are typically used to increase dog activity and improve the dog’s health and care [12]. Thus, these devices have the potential to impact human–dog interactions and ultimately dog welfare.

Actical monitors (Respironics Inc, Murrysville, PA, USA) are externally validated for use with dogs [13] and are currently the most widespread monitors used for dog activity research [3,8,11,14,15,16,17,18,19]. However, Actical monitors currently do not integrate with mobile devices [20]. Two other monitors, the PetPace [11] and Whistle [17], have been evaluated by correlating activity data to the Actical monitors. These studies showed significant correlations of the activity data to Actical monitors; however, these data are likely not as robust as validating the activity monitors directly to the behavioral observation of activity.

For an activity monitor to have a widespread impact on dog health and welfare, it needs to be accurate, affordable, user-friendly, and easily accessible to potential users. The FitBark 2 dog activity monitor (referred to herein as Fitbark; FitBark Inc., Kansas City, MO, USA) which is a 3-axis accelerometer, is affordable, user-friendly, and easily accessible. However, to our knowledge, although they have been used to monitor dog activity in published studies [21,22,23,24], no peer-reviewed studies have externally evaluated FitBark criterion validity. Therefore, the objective of this study was to evaluate FitBark criterion validity by correlating FitBark activity data to dog physical activity, measured via step count. It was hypothesized that FitBark activity monitors would be a valid tool for measuring physical activity in dogs.

## 2. Materials and Methods

### 2.1. Ethical Statement

All experimental procedures were approved by the Truman State University Animal Care and Use Committee. This experiment was conducted in November and December 2019 at Truman State University.

### 2.2. Animals

Pet dogs (*n* = 26; female: *n* = 14; male: *n* = 12) were recruited for this study by advertising to students, staff, and faculty at Truman State University. Participating dogs were given a free item (toy or bone of the dog’s choosing) and entered into a drawing to win a free FitBark activity monitor. Dogs from a variety of breeds, sizes (mean ± SD: 21.8 ± 9.90 kg; minimum: 5.0 kg; maximum: 38.6 kg), and ages (mean ± SD: 3.7 ± 4.25 years; minimum: 8 months; maximum: 15 years) were recruited for the study to increase sample variation (Supplementary Table S1). Upon scheduling, owner consent, dog information including name, sex, birth date, primary breed, spay/neuter status, and any known medical conditions were obtained.

### 2.3. Testing

Dogs were brought to the Truman State University Farm for testing. Upon arrival at the testing facility, each dog was weighed on a non-slip, digital platform scale (W C Redmon Co., Peru, IN, USA). Gait analysis was visually evaluated using the numerical rating scale for visual assessment of gait on a 0–5 scale (0 = clinically sound and 5 = non-weight bearing on a limb while standing or moving) [25]. All dogs included in this study had a gait score of 1 or below.

Each dog’s typical collar was removed and replaced with a flat nylon collar (Vibrant Life, Walmart Inc, Bentonville, AR, USA) with a FitBark monitor attached via zip ties following FitBark collar attachment instructions [26]. Each FitBark monitor was connected via Bluetooth to an iPad (Apple Inc, Cupertino, CA, USA) and had the following information for each dog entered into the FitBark app for the corresponding monitor: name, sex, spay/neuter status, birth date, weight, primary breed, and any known medical conditions. Each collar was adjusted to ensure a snug fit with a two-finger gap between the collar and neck [17,19] and to ensure the FitBark monitor was located ventrally and the D-ring was located dorsally on each dog’s neck.

All testing occurred in an open 12 m × 12 m room with concrete flooring. Four color cameras recording on a DVR system (Amcrest Technologies LLC, Houston, TX, USA) were mounted 1 m from the floor to record dog activity. A fifth camera recorded the time displayed on the iPad that was synced to the FitBark monitor to ensure consistency in recording time between the DVR and the FitBark. Each dog was video recorded and individually tested for 30 min within the facility using a three-phase approach intended to elicit different dog behaviors.

The test began by letting the dog off-leash in the enclosed room. Phase 1 consisted of 10 min for the dog to explore the room off-leash. A dog bed, toys, and a water bowl were present in the room for the dog to interact with. To avoid interaction, the dog owner was not present in the room. A research assistant monitored the dog on a screen connected to the camera system outside of the room. As a result of concerns about separation anxiety, the owner of one dog (Dog 9) was present in the room during phase 1 but had minimal contact with the dog during this phase. Phase 2 consisted of 10 min of human–dog interaction. Phase 2 began when the owner entered the room. The owner was asked to interact with the dog off-leash as he/she typically would, including activities such as petting and playing with the dog. The owner was provided with a chair, dog toys, and treats to use while interacting with the dog. Phase 3 consisted of a 10-min on-leash walk. A nylon leash (1.83 m long, Vibrant Life, Walmart Inc) was provided, and the owner was asked to walk the dog for 10 min. Owners were allowed to choose how they walked the dog, so walking patterns and speed varied. As a result of owner concerns, the owners of two dogs (Dogs 8 and 17) chose to hook the leash to a harness instead of the provided flat collar. Thus, phase 3 data were not analyzed for these dogs. All phases were completed in the same, consecutive order for all dogs.

Following testing of each dog, FitBark data were downloaded at a 1-min epoch length. The video of dog testing was continuously analyzed for step count by one trained observer with an intra-observer reliability of 0.994 (*p* < 0.001; Intraclass Correlation Coefficient, calculated using “irr” R package, RStudio 1.2.5019, Boston, MA, USA). A step was counted every time the front right paw touched the ground. Observations were manually collected using a clicker, and total steps per minute were recorded. If there were any occurrences where the dog was not visible on the cameras (e.g., exited the room), then the data within that minute were excluded from the analysis.

### 2.4. Statistical Analysis

Statistical analyses were performed utilizing Minitab software (Minitab 17.3.1, Minitab LLC, State College, PA, USA). Phase differences in both step count and FitBark activity were determined using Welch’s ANOVA with Games–Howell adjustments (95% confidence). Pearson’s correlation coefficients were calculated to assess the correlation between activity level indicators for the entire dataset as well as at the phase and individual dog level. A correlation coefficient of 0.9–1.0 was considered very strong, 0.7–0.9 was considered high, 0.5–0.7 was considered moderate, 0.3–0.5 was considered low, and 0.0–0.3 was considered negligible [27]. The significance level was fixed at *p* < 0.05.

## 3. Results

**Figure 1:**
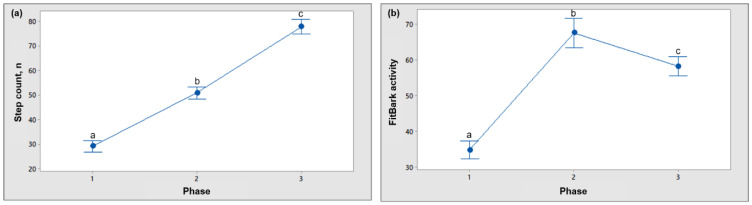
Interval plot showing the 95% confidence interval for the mean of dog step count (**a**) and activity recorded by a FitBark 2 monitor (**b**) across phase 1 (off-leash room explore), 2 (human-dog interaction), and 3 (on-leash walk). Different superscripts indicate significance at *p* < 0.05.

A total of 745 min of video step count and FitBark activity data (referred to as BarkPoints by FitBark Inc.) were analyzed across all 26 dogs. Evaluation of the step count data showed differences between all three phases (*p* < 0.001: Figure 1a) with the step count increasing during each phase of the experiment. Alternatively, evaluation of the FitBark activity data also indicated differences in activity level between all stages (*p* < 0.001; Figure 1b) but with the greatest level of activity during phase 2.

**Figure 2:**
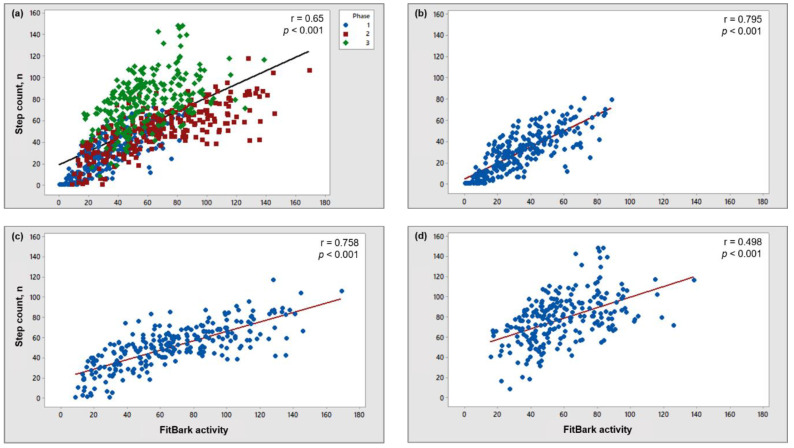
Plot showing the relationship between step count and activity recorded by a FitBark 2 monitor for all dogs across all phases (**a**), phase 1 (off-leash room explore; (**b**)), 2 (human-dog interaction; (**c**)), and 3 (on-leash walk; (**d**)).

Total dog step count and FitBark activity showed a moderate correlation across all phases (r = 0.65, *p* < 0.001; Figure 2a). When evaluating data across each phase individually, a high correlation between step count and FitBark activity was observed during phases 1 (r = 0.795, *p* < 0.001; Figure 2b) and 2 (r = 0.758, *p* < 0.001; Figure 2c) and a low correlation was observed during phase 3 (r = 0.498, *p* < 0.001; Figure 2d).

**Figure 3:**
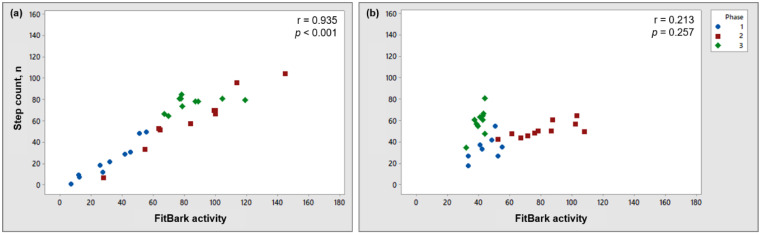
Plot showing the relationship between step count and activity recorded by a FitBark 2 monitor for Dog 25 (an example of a dog showing a very strong correlation; (**a**) and 19 (an example of a dog showing a negligible correlation; (**b**).

When evaluating data for each dog across all phases, the strongest correlations observed between step count and FitBark activity were r = 0.969 (*p* < 0.001; Dog 16) and r = 0.935 (*p* < 0.001; Dog 25; Figure 3a). The weakest correlations observed between step count and FitBark activity across all phases were r = 0.213 (*p* = 0.257; Dog 19; Figure 3b) and r = 0.275 (*p* = 0.156; Dog 10). Examination of the data for individual dogs with poor correlations often found anomalies with FitBark activity during phase 3. While on-leash, many of the dogs showed a relatively low FitBark to step count ratio (Figure 3b) indicating that the FitBark was under-sensing activity. When phase 3 data were removed from these dogs and only phases 1 and 2 were analyzed, the step count and FitBark activity correlation typically improved (e.g., Dog 19: r = 0.796, *p* < 0.001; Dog 10: r = 0.779, *p* < 0.001).

## 4. Discussion

### 4.1. Step Count and FitBark Activity Correlations

Evaluating criterion validity of dog activity monitors is important for increasing access to accurate data for dog owners, veterinarians, researchers, and other animal professionals. Results of the current study were comparable to prior studies validating the Actical accelerometer [13], which has since been widely used to measure physical activity in dogs [3,8,11,14,15,16,17,18,19]. In the current study, high correlations were observed between dog step count and FitBark activity during phase 1 (off-leash room explore; r = 0.795, *p* < 0.001) and phase 2 (human–dog interaction; r = 0.758, *p* < 0.001). This is comparable to the correlations observed between the collar-mounted Actical accelerometer and the distance traveled and time spent moving in dogs (r = 0.89) [13]. While this study did not correlate accelerometer data to distance traveled or time spent moving as done by Hansen and colleagues [13], this study correlated FitBark activity to step count, similar to the methodology used in studies with other animals [28,29,30].

Other studies have correlated Actical accelerometer data to alternative activity monitors including the Actigraph (r = 0.72), PetPace (r = 0.59) [11], and Whistle monitors (r = 0.925) [17]. While the current study did not correlate FitBark and Actical data, the methodology of the current study utilized behavioral observation of step count. Although subject to human error, behavioral observation is a common method used to validate human [31,32,33] and other animal [28,29,30,34,35] activity monitors and should produce more accurate data compared to correlating the device to a previously validated tool.

### 4.2. Three-Phase Approach

This study was unique in that it used a three-phase approach to elicit a range of behaviors in participating dogs. Although step count was the only behavior formally recorded during this study, general dog behaviors were noted during step count analysis. During phase 1, dogs were primarily observed engaging in light-intensity activities such as walking, sitting, and interacting with toys. During phase 2, dogs engaged in light-intensity activities such as walking, sitting, and interacting with toys and vigorous-intensity activities such as running, jumping, and playing with toys. Human–animal interaction was also observed during phase 2, including the owner petting and playing with the dog. During phase 3, dogs were primarily observed engaging in moderate-intensity activity while walking on-leash. The step count analysis showed that dogs took the fewest steps during phase 1, an increased number of steps in phase 2, and the greatest number of steps during phase 3. However, the FitBark data indicated that dogs were more active during phase 2 compared to phase 3.

High correlations were observed between dog step count and FitBark data during phases 1 and 2; however, a low correlation was observed during phase 3. We hypothesize that various on-leash factors impacted the accuracy of phase 3 FitBark activity data such as leash attachment to the collar and modifications to the dog’s gait and stride length. Martin and colleagues [19] reported stronger correlations when a leash was attached to a harness instead of a collar; thus, more research evaluating collar style and attachment location impacts on FitBark activity data is warranted.

### 4.3. Benefits and Drawbacks of the FitBark Activity Monitor

FitBark monitors have multiple benefits that make them a feasible option as a dog behavior and health indicator, intervention, and research tool. FitBark monitors record data through a 3-axis accelerometer, are fairly low cost, and have a battery life of ~6 months. They come in a relatively small size (41 × 28 × 13.5 mm, 10 g), affix to a dog’s collar with zip ties, and are waterproof. Currently, FitBark monitors integrate into an app that syncs the FitBark data for mobile device integration, which is free to users. Data can be viewed online or activity can be downloaded in 1-min epoch lengths. Veterinarian and researcher access to data can also be permitted by the dog owner [36]. Thus, FitBark monitors are affordable, user-friendly, and accessible tools for monitoring dog physical activity.

While FitBark currently has many benefits for users of this tool, we understand that companies can change rapidly. Thus, it is important that this tool continues to be made accessible and user-friendly for a broad population of users. Additionally, as a result of the wide variation in correlations between step count and FitBark data between dogs and activity type, it may be inaccurate to draw wide conclusions between diverse populations of dogs. Instead, understanding what is normal for an individual dog by collecting baseline data before applying a treatment would be a more accurate approach. Additionally, because the step count during the on-leash walk (phase 3) showed a low correlation to the FitBark data, more work evaluating collar style and the leash attachment method should be done to evaluate the best method of collecting activity data for on-leash situations. Prior studies have also identified potential privacy concerns with pet wearable technology [37]. While companion animal wearable technology has the potential to improve the human–animal bond, there are concerns that this may negatively affect it as well [38]. Thus, further evaluation of how these tools can be best used to improve the human–animal bond and dog welfare will be critical.

## 5. Conclusions

Wearable technology is becoming increasingly popular for dogs; thus, it is critical that the information provided to users is accurate. This study showed high correlations between step count and FitBark activity during off-leash situations, indicating that the FitBark 2 monitor is a valid tool for tracking physical activity in off-leash dogs. However, low correlations were observed during on-leash activity; thus, more work should be done to identify the best method of tracking activity in on-leash situations. Because the FitBark 2 is an affordable monitor primarily marketed toward dog owners, this tool, if used correctly, has the potential to make an impact on the health and welfare of dogs.

## Data Availability

The data presented in this study are available on request from the corresponding author.

## Supplementary Materials

The following are available online at https://www.mdpi.com/2076-2615/11/3/781/s1, Table S1. Description of dogs participating in the study.
