# Supplementary material for: Evaluation of the FitBark Activity Monitor for Measuring Physical Activity in Dogs
Source: Animals (Basel). 2021 Mar 11;11(3):781. doi: 10.3390/ani11030781 (PMC7999242; doi:10.3390/ani11030781)
Supplement: Supplementary file 1 [file animals-11-00781-s001.pdf]

Table S1. Description of dogs participating in the study.

Dog ID	Dog Weight (kg)	Dog Age (years)[1]	Gait Score[2]	Sex[1]	Spayed or Neutered (yes/no)[1]	Primary Breed[1]
1	31.75	2	0	M	Y	American Pit Bull Terrier Mix
2	24.04	5	0	F	Y	American Pit Bull Terrier Mix
3	18.59	3	0	F	Y	Australian Cattle Dog Mix
4	29.48	10	1	F	Y	German Shepherd
5	29.02	2	0	M	Y	Boxer
6	21.77	0.7	0	F	Y	Labrador Retriever Mix
7	18.14	0.7	0	F	N	Australian Shepherd
8	29.02	3	0	F	Y	German Shepherd
9	10.88	4	0	F	Y	Australian Shepherd Mix
10	33.11	2	0	M	Y	Goldendoodle
11	4.99	1	0	M	N	Schnauzer (Standard) Mix
12	17.23	14	1	M	Y	Collie (Rough) Mix
13	31.29	1	0	M	Y	Goldendoodle
14	25.85	2	0	F	N	English Shepherd
15	28.12	1	0	M	N	English Shepherd
16	4.99	0.7	0	F	Y	Chihuahua Mix
17	7.26	7	0	F	Y	Boston Terrier
18	38.55	1	0	F	Y	Great Pyrenees
19	36.28	1	0	M	Y	Unknown Mix
20	14.97	1	0	M	Y	Australian Cattle Dog
21	19.50	2	0	F	Y	Labrador Retriever Mix
22	9.52	15	0	M	Y	Lhasa Apso Mix
23	16.78	12	0	F	Y	Unknown Mix
24	9.52	3	0	F	Y	Dachshund Mix
25	25.85	1	0	M	Y	Golden Retriever
26	30.84	2	0	M	Y	Unknown Mix

[1]Attribute identified by the dog owner.

[2] Visually evaluated using the numerical rating scale for visual assessment of gait on a 0-5 scale (0 = clinically sound and 5 = non-weight bearing on a limb while standing or moving). Dogs with a gait score of 2 or above were excluded from the study.
